# Utility of Diagnostic Classification for Children 0–5 to Assess Features of Autism: Comparing In-person and COVID-19 Telehealth Evaluations

**DOI:** 10.1007/s10803-022-05606-y

**Published:** 2022-06-16

**Authors:** Sara Julsrud Holtman, Katherine Skillestad Winans, John D. Hoch

**Affiliations:** 1 College of Social and Behavioral Sciences, Northwest University, 6710 108th Ave NE, Kirkland, WA 98033 USA; 2 Fraser, 3333 University Ave SE, Minneapolis, MN 55414 USA; 3 Department of Educational Psychology, University of Minnesota, Twin Cities, 3333 University Ave SE, Minneapolis, MN 55414 USA

**Keywords:** Autism spectrum disorder, Telehealth, COVID-19, DC: 0–5, Logistic regression, Diagnostic decision making

## Abstract

Logistic regression was used to examine the use of Autism Spectrum diagnostic categories from pre-COVID-19 in-person evaluations and COVID-19 telehealth evaluations at a specialist community mental health clinic. The diagnostic classification for children 0–5 (DC: 0–5) affords a wider range of diagnoses that allowed for inferences of clinician certainty of diagnosis. Use of full criteria diagnoses was significantly lower from telehealth evaluations during the pandemic, and was less certain for younger children, some non-English speakers, and children reporting Native American/Alaska Native race. Higher Child Behavior Checklist (CBCL) ASD subscale scores, lower CBCL total scores, and global developmental delay diagnoses predicted greater use of full ASD diagnoses. Findings suggest factors that could identify children appropriate for telehealth evaluations.

**Supplementary Information:**

The online version contains supplementary material available at 10.1007/s10803-022-05606-y.

There are many obstacles to diagnosing autism spectrum disorder (ASD) in young children. Factors related to geographic location, the limited availability of skilled providers, socioeconomic impacts, and reduced awareness of developmental disorders can impede identification that, in turn, can cause delays in the receipt of early intervention services (Antezana et al., 2017; Bluming et al., 2019). In addition, changes in the observable symptoms of ASD are seen throughout the developmental period as age expectations for social abilities and complexity of movement and play increase (Jones et al., 2014). The COVID-19 pandemic social distancing requirements have created additional barriers to the early identification of ASD and put a spotlight on the growing body of research examining the efficacy of telehealth assessment for young children. The research regarding in-person assessment of ASD has stressed the importance of including measures that evaluate language, cognitive functioning, adaptive skills, and direct-observation, combined with a thorough clinical interview (Huerta & Lord, 2012; Hyman et al., 2020). While some of these measures remain available via telehealth, many are not.

Emerging research suggests the utility of streamlined approaches to the evaluation of ASD that may be applicable to the difficulties of telehealth evaluations (Alfuraydan et al., 2020; Corona et al., 2020; Juarez et al., 2018; Narzisi, 2020; Wagner et al., 2021a, 2021b) and also suggests reducing the burden of comprehensive evaluations may increase access to services. Use of technological advances such as parent recorded video samples combined with developmental history yielded results commensurate with that of more comprehensive in-person evaluations consisting of the Autism Diagnostic Observation Schedule-2^nd^ Edition (ADOS-2), Autism Diagnostic Interview-Revised (ADI-R), and intelligence testing (i.e., the Mullen Scales of Early Learning, Kaufman Brief Intelligence Test; Smith et al., 2017). Other work has supported the reliability of expedited assessment protocols, Sanchez and Constantino (2020) demonstrated that an adaptation of the Childhood Autism Rating Scales-2 (CARS-2) completed by clinicians based only on observations were found to result in reliable diagnoses of ASD when compared to comprehensive evaluations that incorporated the ADOS-2, SRS-2, and clinical interview.

Telehealth evaluations provide for interviews, adaptive skills evaluation, and direct observations. Advances in symptom rating tools suggest possibilities for the future, but as these advances are further validated, clinicians were faced with diagnostic decision making with a new set of constraints at the onset of the COVID-19 pandemic. Research on telehealth ASD evaluations suggests that many parents are comfortable with a telehealth ASD evaluation for their child (Corona et al., 2020; Juarez et al., 2018). Researchers have rapidly deployed telehealth compatible measures of ASD symptoms due to the demands of the COVID-19 pandemic (e.g. TELE ASD PEDS, Wagner et al., 2021a, 2021b; BOSA, Dow et al., 2021). Wagner et al. (2021a) examined provider perceptions of the TELE-ASD-PEDS (TAP), and notably, all providers (n = 9) reported feeling it was appropriate or very appropriate for toddlers to receive a diagnosis of ASD over telehealth. Juarez and colleagues (2018) examined the correspondence of TAP assessments via telehealth with subsequent confirmatory in-clinic ASD evaluations and found that 20% of children that were not diagnosed with ASD over telehealth were identified during in-person evaluations. These false negative cases were predicted by clinician’s self ratings of their confidence in diagnosis.

Telehealth assessment may be a better fit for specific cases. Goldstein and colleagues (2017) conducted a literature review to examine the utility of telehealth assessment related to ASD and found that children with more conventional symptoms of ASD were most suitable for evaluation over telehealth, with more nuanced presentations requiring face-to-face evaluation. Factors that influence the reliability and validity of telehealth psychological assessment included both acceptance and comfort with telehealth for both the patient and provider (Luxton et al., 2014).

Despite these advances in tool development, telehealth solutions are still lacking for many areas of ASD evaluation. Cognitive assessments have been particularly lacking, along with validated replacements for standardized in-clinic measures such as the ADOS-2. This may lead clinicians to use diagnostic categories that reflect less certainty about long-term diagnoses.

## Diagnostic Systems

Diagnostic systems impact how clinicians make sense of interview information and diagnostic tools to arrive at a diagnosis. The Diagnostic and Statistical Manual of Mental Disorders, Fifth Edition (DSM-5; American Psychiatric Association, 2013) requires deficits in both social communication as well as the presence of restricted and repetitive behaviors for the full diagnostic criteria for ASD to be met. The current study evaluates data from a system of care that utilizes the Diagnostic Classification of Mental Health and Developmental Disorders of Infancy and Early Childhood (DC: 0–5; ZERO TO THREE, 2016) in the evaluation of children aged under 6 years of age.

The diagnostic criteria for the full criteria diagnosis of ASD is the same in both systems, however the DC: 0–5 provides options for other diagnoses within the ASD spectrum that are not available in the DSM-5. Also unique to DC: 0–5 is the integration of systemic factors that influence development and perceived psychopathology in young children. This includes use of multiaxial assessment that includes clinical disorders, relational context, physical health conditions and considerations, psychosocial stressors, and developmental competence. This multiaxial assessment may incorporate relevant pandemic-related circumstances and their influences on child symptomatology.

Specific to ASD, the DC: 0–5 system provides the categories of “other neurodevelopmental disorders” (OND), and “early atypical autism” (EA-ASD), which correspond approximately to the DSM-5 categories of “unspecified neurodevelopmental disorder” and the prior DSM-IV diagnosis of “pervasive developmental disorder”. These diagnoses require significant, but subthreshold symptoms, of ASD and thus represent another category on the continuum of ASD spectrum diagnoses. The diagnosis of EA-ASD was developed to allow for the early identification of children who are too young to show complex social communication behaviors, or who have age-typical stereotyped or routinized play (Soto et al., 2016). EA-ASD symptom criteria are the same as those for a diagnosis of full scale ASD under both DSM-5 and DC: 0–5 diagnostic systems, but the DC: 0–5 EA-ASD diagnosis requires fewer qualifying symptoms than full scale ASD diagnosis under either DC: 0–5 or DSM-5. EA-ASD is used for children that meet criteria for at least two deficits in social-communication, one restricted repetitive behavior, and show problems related to their noted symptoms. The DC: 0–5 diagnosis of OND can encompass a range of subthreshold neurodevelopmental deficits in early childhood and is used similarly to the Other Neurodevelopmental Disorders diagnosis in the DSM-5. EA-ASD and OND may be used to mark risk factors that have not yet developed into full ASD, or to mark a lack of clinician ability to observe the symptoms during an evaluation.

The provider data examined here were collected in a state where access to a range of early intervention services can be obtained with private or public insurance with OND, EA-ASD, or ASD diagnoses. While previous research has measured clinician certainty through the identification or dismissal of an ASD diagnosis (McDonnell et al., 2019), OND and EA-ASD describe a middle ground similar to that of the categories of ASD, non-ASD (i.e., PDD-NOS and Aspergers), and non-spectrum as described in Gotham et al. (2007). These diagnoses allow for clinicians to express a degree of uncertainty around diagnoses while being sufficient to access care. In the current study, these diagnoses are conceptualized as measures of clinician certainty or expressions of pragmatic utility of diagnoses to allow for continued treatment or monitoring.

## Confidence and Certainty

Due to the limited test batteries accessible for telehealth testing as compared to in-clinic testing, it may be more difficult to differentially diagnose clients over telehealth and thus affect the use of diagnoses by clinicians. Juarez and colleagues (2018) directly measured clinician confidence in telehealth diagnoses using the clinician confidence scale included in the TAP and found that clinicians were not confident in their ratings for 13% of children evaluated over telehealth. Accuracy of diagnosis and its relationship to confidence was further examined by Hedley and colleagues (2016), who examined clinician confidence and accuracy of diagnosis in the context of telehealth evaluations. Results showed that clinician confidence was predictive of levels of accuracy in the remote assessment of ASD in toddlers with the only significant independent predictor of an ASD diagnosis being parent report of atypical behavior. It is unknown how the ability to observe specific behaviors during telehealth sessions might influence clinician certainty in assigning diagnoses.

## Predictors of Diagnostic Confidence

Although many factors might drive the selection of full criteria ASD compared to partial criteria diagnoses (e.g. EA-ASD or OND), one driving factor might be clinician confidence. McDonnell and colleagues (2019) examined predictors of clinician-rated confidence in diagnosis from in-person ASD evaluations of young children in university-based clinics. The authors used logistic regression to evaluate the relative influence of age, use of private insurance, Vineland Adaptive Behavior Scales, 3^rd^ Edition (VABS-3), IQ scores, ADOS-2 and the CBCL ASD scale. They found that increased child age at the time of evaluation and IQ were associated with reduced diagnostic certainty. The use of private insurance (which was a proxy for increased economic status) was related to increased diagnostic certainty. The authors found other factors related to increased diagnostic certainty, such as the CBCL ASD scale and the ADOS-2 classification of “spectrum” was associated with greater diagnostic certainty. Overall, the findings suggest demographics and rating scale tools are associated with greater clinician confidence in diagnoses.

## Present Study

Much of the existing and emerging research has been produced from university-based research centers with children at high risk of ASD who were specifically referred for an autism evaluation. The present study evaluates the diagnostic decisions of skilled community-based providers trained in the evaluation of early childhood developmental disorders. Prior to the COVID-19, pandemic providers conducted all evaluations in-person, but with the mandated restrictions of the pandemic, the provider conducted all evaluations for children under 6-years old via telehealth. This provided a natural experiment to look at changes in how clinicians used diagnostic categories as an indicator of diagnostic sufficiency and/or diagnostic certainty.

When the provider examined frequencies of diagnostic outcomes in the clinical data, we noted that the rates of EA-ASD and OND diagnoses increased during telehealth use. This led to a research hypothesis that the shift in diagnostic category use might be a proxy for clinician certainty, rather than a reflection of developmental expectations for child behavior. The purpose of this study is to examine predictors of the use of EA-ASD/OND diagnosis compared to full ASD diagnoses in early childhood evaluations from telehealth (pre-COVID) and telehealth (COVID) evaluations. These predictors include demographic categories as well as the use of specific rating scales that were available to the clinicians prior to and during the pandemic.

## Methods

### Procedure

Institutional review board approval was obtained from Institutional review board approval was attained from Northwest University (IRB# 2013) and included the sharing of de-identified data from Fraser Child and Family Center. Data analyzed in this study included all psychological evaluations from children under 6 years of age from January 2018 to March 2021 at Fraser Child and Family Center, a community mental health provider with clinics in the Minneapolis/St. Paul urban core, and the greater metropolitan area. Clients are provided opportunities to opt-out of research use of their data at intake and annually.

Prior to the COVID-19 pandemic, in-clinic evaluations were conducted by a team consisting of a psychologist and a master’s-level clinician (e.g., LICSW, LPCC). Testing appointments were typically scheduled for 3 h. Psychologists generally completed a developmental/cognitive measure (e.g., Bayley-III, Differential Abilities Scales-II, Wechsler Preschool and Primary Scale of Intelligence-IV), observational measure of ASD (e.g., ADOS-2), and caregiver-rating scale of social and interactional skills. Diagnostic assessment/clinical interview was often completed by the master’s level mental health provider. Rating scales, such as the CBCL (Achenbach & Rescorla, 2000), the Autism Spectrum Rating Scale (Goldstein & Naglieri, 2009), and the Strengths and Difficulties Questionnaire (Goodman, 2001) were completed by caregivers.

During the COVID-19 pandemic, telehealth evaluation appointments were scheduled for 3 h with a licensed psychologist. They were conducted using the secure/HIPAA compliant version of the Zoom® video-conferencing software. Families participated from their homes. Psychologists conducted a clinical interview and usually coached parents to complete the TAP (for children under 36 months of age) or adapted situations from the TAP and coached other play-based activities in unscored play-based interactions for children over 36 months of age. These reports and observations were most commonly used to help inform provider ratings on the CARS-2 (Schopler et al., 2010). Psychologists administered behavior rating scales within the telehealth platform, or by securely emailing the link to the caregiver. Rating scales used by the psychologist over telehealth included the VABS-3, ASRS or the Social Responsiveness Scale-2nd Edition (SRS-2), CBCL, and the Developmental Profile-4 (DP-4). Psychologists are trained to clinical reliability in the use of the ADOS-2 and have completed training in the assessment of Autism and other neurodevelopmental disorders, as well as certification in the DC: 0–5 system.

For both in-person and telehealth evaluations, interpreters were provided for families who needed them. For in-person evaluations, 5% of clients required and were provided a licensed interpreter; for telehealth evaluations 7.2% of evaluations used interpreters who joined the telehealth session from a third location.

The diagnostic assessment/clinical interview for all evaluations was guided by a template form in the EMR that requires specific fields of information be entered prior to being finalized. Diagnostic information gathered from these evaluations included DC: 0–5 and ICD-10 descriptors/codes.

### Participants

The sample for this study consisted of 660 participants that met the inclusion criteria based on age (under 6 years) and having scores reported for the ASRS and CBCL. The pre-COVID-19 group consisted of 551 participants, with 109 participants included in the COVID-19 group. The sample was largely male, white, and English speaking. Demographic categories used as predictor variables include child primary language, race, and sex. The demographics of the sample are broadly reflective of the racial and language demographics of the Minneapolis/St. Paul metropolitan area. See Table 1 for details.

**Table 1:** Participant demographics by group and COVID pandemic phase

Demographic variables	During COVID N (%)	Pre-COVID N (%)
Child primary language		
English	99 (90.8)	503 (91.3)
Other	1 (0.9)	26 (4.7)
Somali	5 (4.6)	10 (1.8)
Spanish	4 (3.7)	12 (2.2)
Race		
American Indian or Alaska Native	NA (NA)	11 (2.0)
Asian	1 (0.9)	39 (7.1)
Black or African American	15 (13.8)	62 (11.3)
Other Multiple	11 (10.1)	78 (14.2)
Undisclosed	17 (15.6)	57 (10.3)
White	65 (59.6)	304 (55.2)
Sex		
Female/other	25 (22.9)	106 (19.2)
Male	84 (77.1)	445 (80.8)

A child primary language of “other” is used to identify additional primary languages spoken in the home that are not captured by more a specific language as identified by the client’s self-selection upon intake. For example, Cameroonian Pidgin English would be categorized as “other.”

### Assessment Instruments

Due to the clinical context in which this data was collected, the use of instruments varied across in-person as well as telehealth evaluation contexts. The measures used with high enough frequency across COVID-19 and pre-COVID-19 evaluations are described further below.

#### Child Behavior Checklist (CBCL)

The CBCL for ages 1.5–5 years is a 99-item caregiver report questionnaire that measures a broad range of behavioral and emotional concerns. Items are rated on a Likert scale (0 = Not true, 1 = Somewhat or Sometimes True, 2 = Very True or Often True; Achenbach & Rescorla, 2000). The measure yields three broad domain scores, seven syndrome scales, and five DSM-5 oriented scales. Notably, there is a DSM-5 scale measuring symptoms of ASD. Use of the CBCL in all mental health evaluations for children under the age of 5 years is mandated by state regulations in Minnesota.

#### Autism Spectrum Rating Scale (ASRS)

The ASRS is a caregiver questionnaire that measures behaviors commonly associated with ASD (Goldstein & Naglieri, 2009). The form for children ages two to five years consists of 70 questions rated on a 5-point Likert scale (0 = Never, 4 = Very Frequently) and yield a Total Score, ASRS Scores for Social Communication and Unusual Behaviors, as well as a DSM-5 score. The ASRS DSM-5 score was created to align with symptoms in the DSM-5 manual ASD criteria.

Other assessment instruments used by clinicians, but not analyzed here due to insufficient use include the TAP, VABS-3, DP-4, SRS-2, and the CARS-2. Data for these instruments are summarized in Supplemental Materials Table S1 to provide more description of the study participants.

### Data Analysis Methods

Data extraction and cleaning was performed using the open-source R statistical software (R core team, 2021) and the data.table (Dowle & Arun Srinivasan, 2021), tidyverse (Wickham et al., 2019) packages using the methods previously described in Hoch and Youssef, (2020). The first author extracted ASRS scores from text fields in the medical records and transcribed them into tabular data for analysis. Fifty percent of this tabular data was randomly checked for reliability in data entry, and table joins against the medical records by other staff; no incorrect data was found in this process. If clients had multiple CBCL measures within the study period, only CBCLs completed within 60 days of the evaluation date were used. If clients had multiple evaluations in the study period, only the first evaluation for each client was included in the data set.

Diagnoses were categorized as follows; (a) ASD consisted of any diagnosis of ASD during the evaluation, and (b) subthreshold ASD was any diagnoses of EA-ASD. OND was considered a subthreshold ASD diagnosis if it was provided in the presence of a global developmental delay (GDD) due to the OND category most frequently being used for GDD if ASD was given as a diagnosis. All OND diagnoses were reviewed to verify the text of the clinical summary described the OND diagnosis as being associated with uncertainty around ASD rather than uncertainty about another neurodevelopmental disorder (e.g., ADHD). Clients with other DC: 05 diagnoses were removed from the regression analysis to focus on the differences between full criteria and partial criteria ASD diagnoses.

Binomial regression models testing the influence of demographic variables, evaluation during the COVID-19 pandemic or pre-COVID-19 pandemic, and scores on ASRS and CBCL on certainty of diagnosis were created and tested. Models were selected based on those with the lowest Akaike Information Criteria (AIC; Akaike, 1974). Models with interaction terms (e.g., the interaction of pandemic phase by other tested variables) were rejected due to having higher AIC values. Some measures (CARS-2, TAP) were only available during the pandemic and could not be modeled. VABS-3 scores were extracted but were not available from enough clients during the pandemic to allow the models to converge. Analyzed variables are summarized in Tables 2 and 3.

**Table 2:** Diagnoses use COVID-19 and Pre-COVID-19

Diagnoses	During COVID N (%)	Pre-COVID N (%)
Developmental delay		
Global developmental delay	31 (28.4)	283 (51.4)
No global developmental delay	78 (71.6)	268 (48.6)
Language delay		
Language delay	NA (NA)	66 (12.0)
No language delay	109 (100)	485 (88.0)
ASD clear/unclear		
ASD	71 (71)	340 (81.1)
EA-ASD/OND	29 (29)	79 (18.9)
ASD ordinal		
ASD	71 (55.5)	340 (57.1)
EA-ASD/OND	29 (22.7)	79 (13.3)
NoASD	28 (21.9)	176 (29.6)

**Table 3:** Rating scale measures by diagnosis and COVID-19 pandemic phase

Measure	Subthreshold *m* (SD)	Subthreshold N	ASD *m* (SD)	ASD N
Pre-COVID-19				
ASRS DSM-5	66.13 (11.69)	79	72.13 (9.51)	339
ASRS Social Comm	64.89 (9.36)	79	70.19 (7.27)	340
ASRS Unusual Beh	60.34 (11.94)	79	63.33 (10.63)	340
CBCL ASD DSM-5	68.92 (11)	79	73.16 (9.83)	340
CBCL total	63.68 (14.29)	79	63.99 (12.02)	340
During COVID-19				
ASRS DSM-5	68.93 (9.67)	29	74.22 (6.44)	71
ASRS Social Comm	67.55 (8.21)	29	70.14 (5.18)	71
ASRS Unusual Beh	61.66 (8.75)	29	66.77 (9.67)	71
CBCL ASD DSM-5	69.17 (9.58)	29	74.75 (7.90)	71
CBCL total	64.41 (10.78)	29	67.04 (10.10)	71

Log Odds Ratios, the increase or decrease in probability of a certain diagnosis are used to represent the results of regression analysis. For example, an odds ratio of two would indicate a twofold increase in the probability of a certain ASD diagnosis for each unit increase in the predictor variable (e.g., moving from pre-COVID-19 to COVID-19). Negative odds ratios index a decreased likelihood of full ASD diagnoses. The most prevalent values: *White*,* English*,* Male*, were used as comparison categories to describe the relative likelihood of a full ASD vs. OND/EA-ASD diagnoses.

Logistic regression was used to test the hypothesis of the study, that the use of diagnoses reflecting certainty of diagnosis of ASD differed between COVID-19 and pre-COVID-19 evaluations. The secondary hypothesis tested in the regression models was that certainty would be related to the use of diagnostic rating scales (e.g. CBCL, ASRS). To control for possible changes in demographics of the clients who attended evaluations prior to COVID-19 and during COVID-19 time periods, we entered demographic variables including, child age, race, other diagnoses, and primary language. We also used the analysis of demographic variables to reveal subgroups for whom clinicians show greater or reduced diagnostic certainty.

## Results

### Descriptive Results

When examined together, rates of ASD and OND/EA-ASD diagnoses increased slightly during the COVID-19 Pandemic. Pre-COVID 70% of clients evaluated received an ASD or OND/EA-ASD diagnosis compared to 77.6% during the pandemic. This difference was not significant in a Pearson Chi Squared test that tested the probability of any ASD diagnosis compared to no ASD diagnosis between pre-COVID-19 and COVID-19 pandemic evaluations (Chi Squared = 3.41, SE(COVID) = 1.28, SE(Pre-COVID) = 1.83, p < 0.07). However, the use of full ASD compared to OND/EA-ASD reduced greatly during the pandemic. Diagnostic variables by pandemic phase are shown in Table 2 below.

Scores on parent and clinician completed rating scales during the pandemic and prior to the pandemic are shown in Table 3 below.

### Diagnostic Certainty and Demographics

Use of logistic regression indicates that the use of telehealth during COVID-19 compared to Pre-COVID-19 in person assessments was a significant predictor of certainty of diagnosis as indexed by use of full criteria compared to partial criteria diagnostic categories. Pre-COVID-19 evaluations were more likely to result in full criteria diagnoses (Log Odds Ratio (OR) 1.81, Standard Error (SE) = 0.3, t = 2, p < 0.05, 95% Confidence Interval (CI) [1.01, 3.25]).

The most important demographic variables to predict diagnostic certainty were age at diagnosis, global developmental delay, and race. A greater child age at the time of diagnosis (OR 1.03, SE = 0.01, t = 2.57, p < 0.05, CI [1.01, 1.05]) was associated with greater likelihood of full criteria diagnosis. The presence of Global Developmental Delay diagnosis (OR 2.46, SE = 0.28, t = 3.21, p < 0.001, CI [1.42, 4.25]) was associated with increased use of full ASD diagnoses. Having a primary language categorized as “Other” was associated with a full ASD diagnosis (OR 5.48, SE = 0.86, t = 1.97, p < 0.05, CI [1.01, 29.62]). Other demographic categories were associated with reduced certainty of diagnosis; having a race of Native American/Alaska Native (NA/AN) was associated with reduced use of full ASD diagnoses (OR 0.09, SE = 0.96, t = -2.54, p < 0.05, CI [0.01, 0.57]). Language delay diagnoses, other languages, and races were not associated with differences in likelihood of assigning full criteria vs. partial criteria diagnoses.

### Diagnostic Certainty and Clinical Scales

**Fig. 1:**
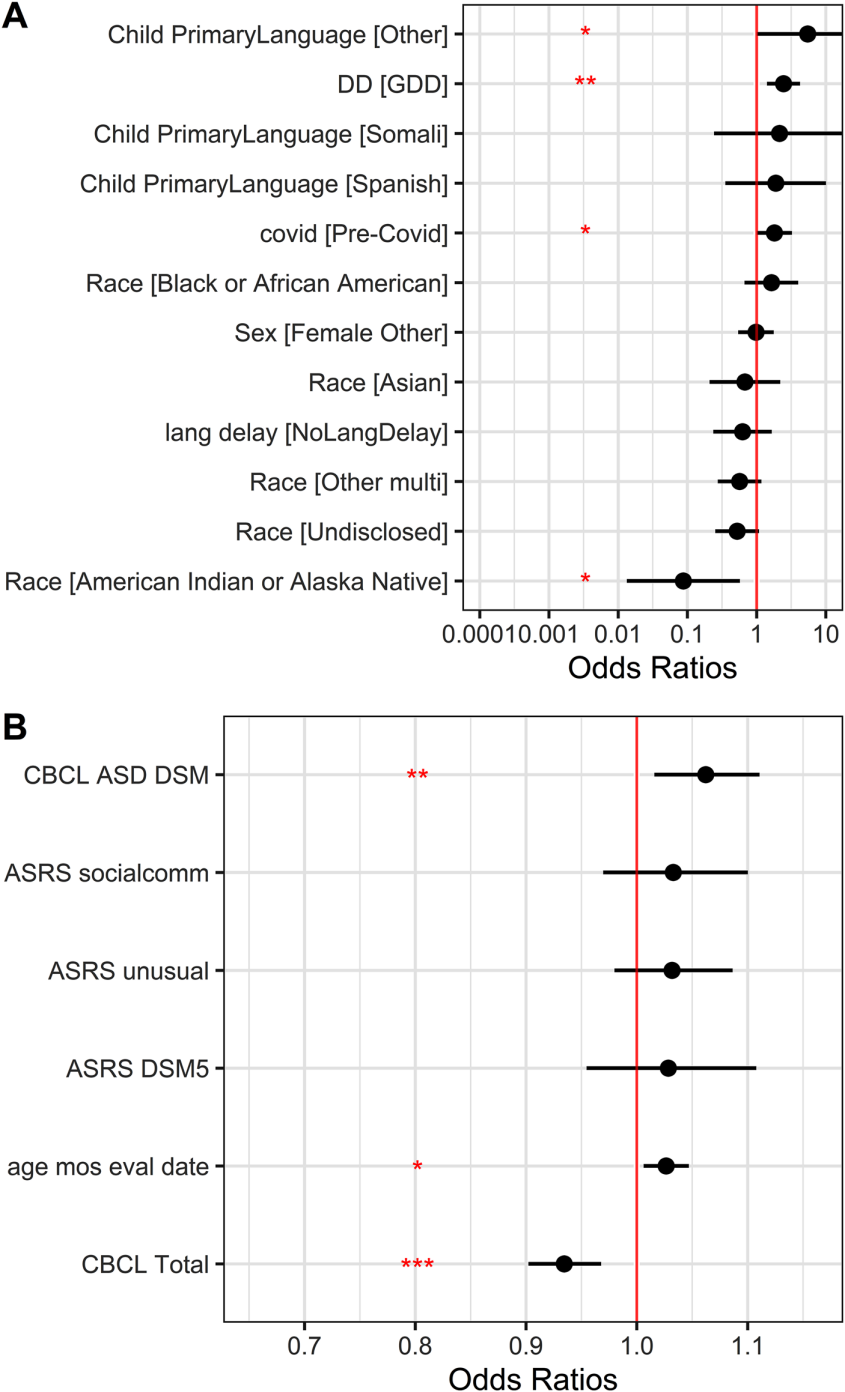
Binomial model. * = p<.05, ** = p<.01, *** = p<.001. Panel **A** Categorical Variables: DD=Developmental Delay, GDD=Global Developmental Delay, Pre-Covid = Evaluation conducted prior to 3/15/2020, Sex Female Other= any sex identification that was not Male, Lang delay=Language Delay, Race Other Multi= Race that included Multi-racial, client entered race, or categories with too few clients to analyze, Primary Language [Other]= Language categories with too few clients to analyze. Panel **B** Quantitative Variables: CBCL ASD DSM=Child Behavior Checklist Autism Spectrum Diagnostic and Statistical Manual Subscale, ASRS socialcomm=Autism Spectrum Rating Scale Social Communication Subscale, ASRS unusual= Autism Spectrum Rating Scale Unusual Behaviors Subscale, ASRS DSM5= Autism Spectrum Rating Scale Diagnostic and Statistical Manual 5 Subscale, age mos eval date= child age in months at date of evaluation, CBCL Total=Child Behavior Checklist Total Score

Rating scales that were associated with differences in use of full ASD compared to EA-ASD/OND diagnosis were the CBCL ASD DSM-5 subscale (OR 1.06, SE = 0.02, t = 2.65, p < 0.01, CI [1.02, 1.11]). The CBCL Total score was associated with use of partial criteria diagnoses (OR 0.93, SE = 0.02, t = − 3.78, p < 0.001, CI [0.9,0.97]); such that higher CBCL total scores corresponded to more likelihood of use of EA-ASD/OND diagnoses. The ASRS DSM-5 score, and Unusual Behavior and Social Communication subscales were not significantly associated with use of full ASD diagnoses compared to partial criteria diagnoses. See Fig. 1 for outcome of the Logistic Regression model.

## Discussion

We found a number of factors to be associated with an increased likelihood of receiving a full criteria ASD diagnosis. Because we conceptualized full criteria ASD diagnoses as reflecting greater clinician confidence within the affordances of the DC: 0–5 diagnostic system, we are able to examine the relative influence of the pandemic, demographic categories, and rating scale variables on clinician certainty of ASD diagnoses within a community provider.

In this sample, use of full criteria diagnoses dropped during the COVID-19 pandemic with the use of telehealth as evidenced by the increased application of EA-ASD and OND diagnoses as compared to that of full criteria ASD. This suggests that clinicians responded to the pandemic and changes to telehealth by adapting the diagnoses they used.

Other factors remained important to diagnostic decision making both pre-COVID-19 in-person evaluations and during the COVID-19 pandemic telehealth evaluations. Age at evaluation was an important predictor of use of full criteria diagnoses within the age range of the DC: 05 system studied here, with older children being more likely to show full criteria of ASD diagnoses. This may be due to these children showing symptoms that are more easily observed over telehealth, or might be due to the increased availability of collateral reporters such as early childhood educators in this age range. This finding contrasts with the findings of McDonnell and colleagues (2019), who found that increased age predicted reduced clinician confidence. The age range of the present study and that of the children evaluated in the McDonnell study overlap, with a similar mean age in the sample. It is unclear whether clinician ratings of confidence captured as used in the McDonnel et al. study captured different aspects of confidence than the use of subthreshold diagnoses which was hypothesized as a possible proxy for confidence in the current study.

The CBCL DSM-ASD scale was the best measure for predicting use of full criteria ASD diagnosis, and the CBCL Total Score was a good negative predictor of full criteria diagnostic confidence. This finding suggests that clinicians are less confident in providing ASD diagnoses in the presence of high levels of other internalizing or externalizing behavior reports. The predictive value of the CBCL-ASD scale was higher than that of the ASRS summary score or subscales in this data set, a finding that should be further examined in non-clinical populations. The predictive value of the CBCL-ASD subscale is coherent with the findings of McDonnell and colleagues (2019) and appears to be highly associated with confidence in diagnosis across both in-person and telehealth evaluations. This measure remained robust against statistically controlled differences in demographic categories and pre-COVID compared to COVID evaluations were included in the model.

Other diagnoses were important in predicting the use of full criteria ASD diagnoses. The presence of GDD increased clinician confidence in ASD diagnosis, but confidence was unaffected by the presence or absence of a language delay diagnoses.

Demographic categories such as speaking non-English primary languages, or having a race of NA/AN predicted lower clinician certainty of diagnosis. Child primary use of Spanish and Somali language were reported at sufficient numbers to allow analysis and were not found to significantly predict clinician confidence in diagnosis. However, the category that included less common, or multiple non-English languages was associated with reduced confidence compared to English speakers. This finding may relate to the lack of available skilled interpreters in less common languages, or due to the lack of translated rating scales available for use or could be due to other factors. The finding that confidence was lower for the NA/AN group bears future investigation. It is based on the results of n = 11 participants which suggests further study and replication. This difference may reflect differences in cultural expectations for children compared to the diagnostic criteria for ASD, or may reflect other social or clinician/family cultural differences. Although there is limited literature specific to ASD in this population, these results are in line with Dyches et al. (2004) findings that NA/AN children were two-times less likely to be provided services for ASD under IDEA than that of their Black and Asian/Pacific Islander counterparts. Evidence suggests that NA/AN children are also underrepresented in access to medical and mental health based ASD services (Bilaver et al, 2021). Our findings add to a very small empirical literature base on Native American/Alaska Native experiences in ASD evaluations.

### Strengths

The use of extant data from a community provider allows for an understanding of real-world decision making outside the confines of university affiliated clinics or research studies where assessment development often occurs. The study takes advantage of the natural experiment brought on by COVID-19 restrictions and the availability of DC: 0–5 diagnoses that allow for access to services despite varying degrees of diagnostic information sources at the time of diagnosis. Additionally, the use of DC: 0–5 allows for integrated conceptualization of pandemic-related factors such as parent stress levels and the quality of the parent–child relationship. The use of logistic regression models allows for a mix of categorical and quantitative predictors and prevents the issues of multiple hypothesis testing false positive findings associated with creating multiple separate regression models.

### Limitations

The current research was completed based on extant data from a clinical evaluation. As such, it is unknown the degree to which various measures informed actual clinical decision making. For example, in some cases, the results of the CBCL may have been immediately available to clinicians, or this may have been provided after the clinician had already provided diagnostic feedback to a client.

The association of GDD diagnosis with certainty around ASD diagnosis may be due to the need for additional measures to diagnose GDD rather than the presence of the disorder itself. Children diagnosed with GDD under the DC: 05 system require an adaptive behavior and cognitive assessment delivered as part of the evaluation or reviewed from prior testing. As the pandemic continued, both school districts and the provider were unable to conduct in-person cognitive testing, possibly resulting in reduced use of GDD. In terms of the current study, the presence of GDD may have been a proxy for a greater breadth of records and testing available for review which may have led to greater use of full criteria diagnoses.

Many changes to clinical practice occurred between the COVID-19 and pre-COVID-19 phases examined in this study. These included the change from in-person to telehealth evaluations, but also a change from team evaluations to individual evaluations. Prior to COVID-19, clinicians conducted child observations in clinic, not in the home setting. In addition, during the pandemic, combined speech, occupational therapy, and psychological evaluations were suspended resulting in reduced availability of language and motor delay information at the time of diagnosis compared to pre-COVID-19. During the pandemic, the provider greatly increased the access to care for rural residents and it is unknown how this population may have affected diagnostic certainty.

Parent, child, and clinician stress levels are likely to be very different prior to COVID-19 compared to during COVID-19 and it is unknown how these other changes may have impacted clinical decision making. For example, Italian mothers' pandemic-realted stress level mediated the relationship between their individual stress and that of sympoms of depression in their child (Babore et al., 2021). The American Psychological Association (2021) reports increased levels of parenting stress and difficulties with decision making which are higher than pre-COVID-19 levels. Of note, the best model fit for the current data did not show differences in the influence of measures during COVID-19 compared to pre-COVID-19, so it is likely that the relative value of these measures and demographic factors remained the same.

Diagnostic certainty assessed via categories of diagnoses is a blunt measure of a nuanced concept. The category of EA-ASD was not developed to reflect certainty of diagnosis, but rather to allow for increased sensitivity to allow identification of children who are showing some aspects of ASD. Because use of OND/EA-ASD diagnoses varied with the onset of the pandemic and use of telehealth, while total use of full ASD diagnoses and non-ASD diagnoses did not vary, it is most likely that the differences in use were due to clinician certainty rather than changes in child development. The Clinician Confidence scale of the TAP provides one measure that may be useful in future research on clinician confidence. McDonnell et al, (2019) used clinician self-ratings of diagnostic confidence on a four-point scale during in-person evaluations which may provide another measure to apply to telehealth diagnostic evaluations.

There were limitations to the demographics available in this data set due to its analysis as an extant data set. There are no data available that describe household income as a demographic variable in this data set. Past analyses of home addresses against U.S. census data suggest poverty levels and household incomes at the provider are reflective of the general population of the service area. The finding that decreased certainty was associated with clients who self-selected their race as being NA/AN is important to assess further. Although this result was significant, it was a small effect and based on a small total sample. The sample included sufficient numbers of other language and racial groups to reliably estimate differences between these groups and the reference group (most common group) of English speaking, White males. The male/female skew of the sample is reflective of levels typically seen in clinics who see are large proportion of clients for whom ASD is suspected. The sample is otherwise demographically reflective of the Minneapolis urban area where the provider is located. The generalizability may be limited for areas with differing demographics.

### Future Research

Direct evaluation of clinician confidence using scales such as the confidence scales in the TAPS or those in video-based measures (e.g., Naturalistic Observation Diagnostic Assessment; Smith et al, 2017) may provide a direct test of the level of clinician confidence based on demographic categories, telehealth use, and information from diagnostic and screening tools. Experimental studies could be designed to determine which information allows clinicians to make confident and accurate diagnostic decisions. One example of a novel study was used clinician impression from the first few minutes of a diagnostic in person encounter to determine whether first impressions held up to full evaluation results in the diagnosis of ASD (Wieckowski et al., 2021). These findings suggest that initial impressions may play an important role in diagnosis and may influence clinician confidence. These findings should be examined in telehealth settings. Additionally, future research could review/compare telehealth evaluations results with children that are recommended to participate in in-clinic testing. The significant association of the CBCL ASD DSM-5 subscale with full diagnoses as compared to the lengthier ASRS scales of DSM-5 scale, and its Social Communication, and Unusual Behaviors subscales warrants more detailed exploration in future work.

### Clinical Implications

Based on this work, experienced clinicians should consider cultural and linguistic factors carefully when providing diagnoses due to the finding that diagnostic certainty was lower for Native American/Alaska Native clients, and for native speakers of some non-English languages. The predictive value of the CBCL, both as an indication of possible other diagnostic needs in the presence of a high Total Score, as well as use of the DSM-5 ASD subscale as an indicator of ASD was supported by this study. While clinicians were less likely to give full ASD criteria diagnoses during the COVID-19 pandemic, they continued to provide diagnoses that indexed a concern for ASD that provided access to early intervention through the flexibility allowed in the DC: 05 diagnostic framework.

## Supplementary Information

Below is the link to the electronic supplementary material.

Supplementary file1 (DOCX 18 kb)
