# Supplementary material for: Utility of Diagnostic Classification for Children 0–5 to Assess Features of Autism: Comparing In-person and COVID-19 Telehealth Evaluations
Source: J Autism Dev Disord. 2022 Jun 16;52(12):5114–25. doi: 10.1007/s10803-022-05606-y (PMC9200933; doi:10.1007/s10803-022-05606-y)
Supplement: Supplementary file 1 — Supplementary file1 (DOCX 18 kb) [file 10803_2022_5606_MOESM1_ESM.docx]

**Supplemental Materials**

*Additional Rating Scale Measures by Diagnosis and COVID-19 Pandemic Phase*

| Measure | Subthreshold  *m*(SD) | Subthreshold  N | ASD  *m*(SD) | ASD  N |
| --- | --- | --- | --- | --- |
| **Pre-Covid** | | | | |
| CARS T score | NA (NA) | NA | 42 (NA) | 1 |
| CARS Raw Score | 26 (2.83) | 2 | 34.33 (2.86) | 5 |
| Vineland-3 ABC SS | 73.2 (9.26) | 54 | 64.52 (12.44) | 194 |
| Vineland-3 Comm SS | 70.48 (14.43) | 54 | 58.88 (20.43) | 194 |
| **During Covid** | | | | |
| CARS T | 44.00 (12.32) | 7 | 48.25 (8.51) | 8 |
| CARS Raw Score | NA (NA) | NA | 35.40 (2.99) | 5 |
| TELE ASD PEDS Forced Choice | 2.57 (0.53) | 7 | 2.00 (0.0) | 8 |
| TELE ASD PEDS Total Score | 14.14 (4.26) | 7 | 15.00 (3.34) | 8 |
| Vineland-3 ABC ss | 68.82 (10.98) | 11 | 66.89 (10.58) | 46 |
| Vineland-3 Comm ss | 63.09 (18.17) | 11 | 61.64 (19.88) | 46 |
| TELE ASD PEDS Forced Choice | 2.57 (0.53) | 7 | 2.00 (0.0) | 8 |
| TELE ASD PEDS Total Score | 14.14 (4.26) | 7 | 15.00 (3.34) | 8 |
